# Bibliographical

**Published:** 1895-11

**Authors:** 


### Bibliographical.

A Manual of Chemistry. A guide to lectures and laboratory work for beginners in chemistry. A text-book specially adapted for students of Medicine, Pharmacy and Dentistry. By W. Simon, Ph. D., M. D., Professor of Chemistry in the College of Physicians and Surgeons of Baltimore, in the Maryland College of Pharmacy and in the Baltimore College of Dental Surgery. Fifth Edition. Thoroughly revised. With forty-four illustrations, and eight colored plates representing sixty-four chemical re-actions.

This edition has been prepared with great care so that it now meets the needs of the student better than ever before; many changes and additions have been made, such as have been suggested by the general advance of the science and the enlarged experience of the author.

The work is presented in seven parts and arranged in such a manneras to lead the student on in a progressive method, from the more elementary subjects to those more advanced and complex.

The arrangement and presentation of the subject is most admirable and is well adapted to the needs of the student. Chemistry, as here presented, will certainly not be the dry and uninteresting study it is so often thought to be.

The author is thoroughly fitted for the preparation of such a work, not only by his familiarity with the subject, but from his experience as a teacher; thus learning the real needs of the student and the difficulties he has to meet.

The work is one that we can heartily commend to all dental students, as one especially adapted to their requirements. Every dentist, who aspires to the possession of a library, should have this volume, at least for reference, if not for study.

The work is published by Lea Brothers & Co., of Philadelphia, and is obtainable of any bookseller.

